# *Rickettsia* Detected in the Reptile Tick *Bothriocroton hydrosauri* from the Lizard *Tiliqua rugosa* in South Australia

**DOI:** 10.3390/pathogens5020041

**Published:** 2016-06-08

**Authors:** Harriet Whiley, Georgie Custance, Stephen Graves, John Stenos, Michael Taylor, Kirstin Ross, Michael G. Gardner

**Affiliations:** 1 School of the Environment, Health and the Environment, Flinders University, GPO BOX 2100, Adelaide 5001, Australia; harriet.whiley@flinders.edu.au (H.W.); michael.taylor@flinders.edu.au (M.T.); kirstin.ross@Flinders.edu.au (K.R.); 2 School of Biological Sciences, Flinders University, GPO Box 2100, Adelaide 5001, Australia; cust0014@flinders.edu.au; 3 Australian Rickettsial Reference Laboratory, Barwon Health, Geelong Hospital, PO BOX 281, Geelong 3220, Australia; graves.rickettsia@gmail.com (S.G.); JOHNS@barwonhealth.org.au (J.S.); 4 Evolutionary Biology Unit, South Australian Museum, North Terrace, Adelaide 5000, Australia

**Keywords:** *Rickettsia*, Flinders Island spotted fever, rickettsioses, *Bothriocroton hydrosauri*, *Tiliqua rugosa*, tick, PCR

## Abstract

Rickettsiosis is a potentially fatal tick borne disease. It is caused by the obligate intracellular bacteria *Rickettsia*, which is transferred to humans through salivary excretions of ticks during the biting process. Globally, the incidence of tick-borne diseases is increasing; as such, there is a need for a greater understanding of tick–host interactions to create more informed risk management strategies. Flinders Island spotted fever rickettsioses has been identified throughout Australia (Tasmania, South Australia, Queensland and Torres Strait Islands) with possible identifications in Thailand, Sri Lanka and Italy. Flinders Island spotted fever is thought to be spread through tick bites and the reptile tick *Bothriocroton hydrosauri* has been implicated as a vector in this transmission. This study used qPCR to assay *Bothriocroton hydrosauri* ticks collected from *Tiliqua rugosa* (sleepy lizard) hosts on mainland South Australia near where spotted fever cases have been identified. We report that, although we discovered *Rickettsia* in all tick samples, it was not *Rickettsia honei.* This study is the first to use PCR to positively identify *Rickettsia* from South Australian *Bothriocroton* *hydrosauri* ticks collected from *Tiliqua rugosa* (sleepy lizard) hosts. These findings suggest that *B. hydrosauri* may be a vector of multiple *Rickettsia* spp. Also as all 41 tested *B. hydrosauri* ticks were positive for *Rickettsia* this indicates an extremely high prevalence within the studied area in South Australia.

## 1. Introduction

Worldwide, the incidence of tick borne disease is increasing [1]. This includes tick borne rickettsiosis which is caused by the obligate intracellular bacteria belonging to the *Rickettsia* genus [2]. Rickettsiosis in humans is a potentially fatal febrile illness that can be categorised as scrub typhus, typhus group and spotted fever group rickettsioses [3]. Presently, 16 of the 25 known *Rickettsia* species have been identified as human pathogens [4]. *Rickettsia* are present in the tick salivary excretions and transferred to humans during the tick biting process [3]. In Australia, Flinders Island spotted fever is an Australian tick borne disease caused by *R. honei* (also known as strain TT-118). It was first discovered on Flinders Island, Tasmania, but has since been detected in clinical patients from other parts of Australia, including in South Australia [5,6]. There has also been a reported cases of Flinders Island spotted fever in Nepal [7]. *Rickettsia honei* has also been detected in Thailand from larval *Ixodes* and *Rhipicephalus* ticks and *I. granulatus* from *Rattus rattus* and in Texas, USA, from *Amblyomma cajennense* ticks taken from cattle. However, the human pathogenicity of *R. honei* in Thailand and USA has not been confirmed [8].

Previously, Stenos *et al.* [9] examined reptile ticks *Bothriocroton hydrosauri* from Flinders Island, Australia, and detected *Rickettsia* via PCR in 63% (29/46) ticks. Subsequent sequencing of seven cell culture samples confirmed a 100% match to *R. honei*. The ticks had been collected from reptile hosts including 12 *Tiliqua nigrolutea* (blue tongue lizards), four *Agkistrodon contortic* (copperhead snakes) and three *Notechis scutatus* (tiger snakes). All blood samples collected from reptile hosts were negative for *Rickettsia*.

In South Australia, there have been confirmed cases of *R. honei* isolated from patients in the same geographical location known for *B. hydrosauri* [10]. However, *R. honei* has not been previously identified from South Australian *B. hydrosauri* but is considered likely to occur in this tick species due to the proximity of the disease and the ticks. This study used PCR to test for the presence of *Rickettsia* in *B. hydrosauri* collected from *Tiliqua rugosa* (the sleepy lizard) hosts found in the South Australian Murray Mallee (SAMM) region.

## 2. Results and Discussion

All of the 41 *B. hydrosauri* ticks tested were positive for *Rickettsia* DNA. We suspected the species would be *R. honei*, however, all of the eight sequenced *ompA* products (KX290293-KX290300) had 99% homology with an undescribed species (accession number: EU283837). The closest match with a named species was 95% with *Rickettsia tamurae* (accession number: DQ103259.1). Using PCR, the DNA from the 15 *T. rugosa* hosts blood samples were all negative.

This study is the first to positively identify *Rickettsia* from South Australian *B. hydrosauri* ticks but instead of the expected agent of Flinders Island Fever, *R. honei* we uncovered potentially a different, as yet uncharacterized, species. Our study has several important findings. First, it indicates that the reptile tick *B. hydrosauri* is possibly not the host of *R. honei* in the sampled area. Second, our results implicates *B. hydrosauri* as a potential vector of other species of *Rickettsia* and not just *R. honei.* Previous studies have only identified *Rickettsia* from *B. hydrosauri* ticks collected from Flinders Island, Tasmania [9].

Third, our findings indicate an extremely high prevalence of *Rickettsia* within the studied area in South Australia as all ticks tested were positive. Reports from Stenos [9] indicate a lower prevalence on Flinders Island, albeit of a different *Rickettsia*.

A forth outcome is our lack of detecting horizontal transmission of *Rickettsia* from tick to lizard and our results support vertical transmission although they do not exclude horizontal transmission being present. We did find that all life stages: adult, larval, and nymph; tested positive for *Rickettsia*. Transovarial transmission has been demonstrated for some *Rickettsia* species [11]. The presence of *Rickettsia* in the *B. hydrosauri* larvae further supports this mode of transmission. This supports the findings by Stenos *et al.* [9] that did not detect *R. honei* from any reptile hosts carrying *Rickettsia* positive ticks. The negative results could be due to the numbers of *Rickettsia* present in the blood sample being less than the limit of detection or that *T. rugosa* is not a main host. Further work on tissues would be required to determine if *Rickettsia* remain viable, but quiescent, in any organ of the lizards.

Although further work would be required to identify if this species of *Rickettsia* causes disease, as *B. hydrosauri* are known to feed on humans [12], our results indicate a greater potential for humans to be exposed to a potential vector of disease than previously thought. This reptile and tick association occurs across a large area of Southern Australia, particularly in South Australia in rural areas close to Adelaide. Further work would be required to understand if the prevalence in the studied area is mirrored elsewhere in the ticks range in South Australia or in other tick species or indeed if the same species of *Rickettsia* is involved in different parts of the tick and lizards range. Future studies comparing these samples to clinical isolates would provide insight into the epidemiological significance of these results and how the prevalence of *Rickettsia* in *B. hydrosauri* hosts affects the incidence of spotted fever rickettsiosis in humans.

Finally, we have potentially uncovered an unnamed species of *Rickettsia* which has previously only been reported from the reptile tick *Amblyomma fimbriatum* [13] from the Northern Territory of Australia, thus extending the range and potential vectors of this *Rickettsia*. Confirmation of this species would require isolation and further genomic characterisation. The disease causing nature of this species also needs to be confirmed.

## 3. Experimental Section

### 3.1. Sample Collection

We utilized 35 adult male, 1 adult female, 3 nymphs and 2 larval *B. hydrosauri* ticks harvested from 35 *T. rugosa* hosts captured from the South Australian Murray Mallee (SAMM) region during a previous study [14]. Sampling was conducted in the hot and dry summer months of November to February on days when the ground temperature was <28 °C. We also sampled blood from the caudal vein of a subset of 15 lizard hosts.

### 3.2. DNA Extraction

DNA was extracted from 41 whole ticks using the PowerSoil^®^DNA Isolation Kit MO BIO Laboratories, Inc., Carlsbad, CA, USA) following manufactures instructions, additionally incorporating homogenization by 0.1 mm zirconium bead-milling to disrupt bacteria. DNA extraction from the blood of lizards followed [14].

### 3.3. PCR Detection

**Figure 1:**
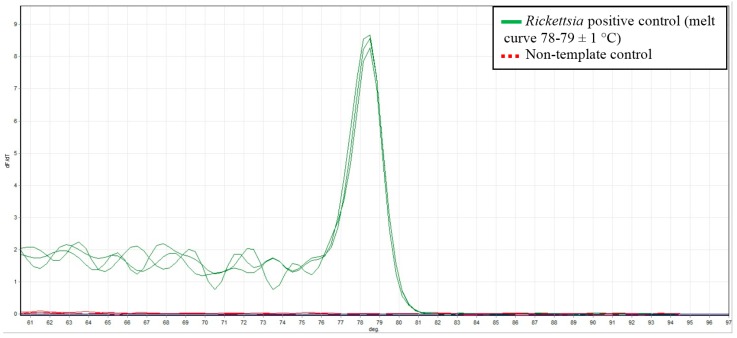
Melt curve data showing the *Rickettsia* positive control with a T*_m_* of 78–79 ± 1 °C (solid green line) and the no- template control (dashed red line).

*Rickettsia* specific qPCR was performed using the Rotor-Gene^®^ SYBR^®^ Green PCR Kit (Qiagen, Victoria, Australia) and previously described primers [15]. All samples were tested in duplicate. The 25 μL reactions contained 12.5 μL of Rotor-Gene SYBR Green PCR Master Mix, 5 μL RNase-Free Water, 1 μM CS-F (5′-TCG CAA ATG TTC ACG GTA CTT T-3′) , 1 μM CS-R (5′-TCG TGC ATT TCT TTC CAT TGT G-3′) and 2.5 μL of template DNA (ca. 20 ng). The cycling conditions included an initial hold at 95 °C for 5 min, followed by 40 cycles consisting of 95 °C for 5 s and 60 °C for 10 s. All PCR reactions were carried in a RotorGene 3000 (Corbett Research, Sydney, Australia) with data acquisition at 60 °C on the Green channel (excitation at 470 nm, detection at 510 nm) at a gain of 9.67. Melt curve data was also acquired on this channel using a ramping rate of 1 °C/90 s from 60 °C to 95 °C. Each PCR run included a positive *R. honei* control (Australian Rickettsial Information Laboratory in Geelong, Victoria, Australia) and a non-template control of nuclease free water (Qiagen, Australia). For each reaction the melt curve was analysed and a positive *Rickettsia* result was confirmed with a melting temperature (T*_m_*) of 78–79 ± 1 °C as shown in Figure 1.

### 3.4. Sequencing of Positive Samples

We sequenced the PCR products from a subset of 10 positive tick samples to identify the *Rickettsia* species amplified. PCR products were purified and sequenced by the Australian Genome Research Facility Ltd (Adelaide, Australia) using previously described primers CS-F (5′-TCG CAA ATG TTC ACG GTA CTT T-3′) and CS-R (5′-TCG TGC ATT TCT TTC CAT TGT G-3′) [15]. After a positive match with *Rickettsia* with this small fragment, the samples were amplified with primers Rr190.70p (ATG GCG AAT ATT TCT CCA AAA) and Rr190.602n (AGT GCA GCA TTC GCT CCC CCT) [16] and eight PCR products were successfully sequenced. All sequences were analysed using the Basic Local Alignment Search Tool (BLAST) [17].

## 4. Conclusions

This study provides an insight into the prevalence and variety of Rickettsioses in South Australia. Intriguingly, we did not uncover the causative agent of Flinders Island spotted fever, *R. honei*, but possibly a yet unnamed species. This study was the first to identify *Rickettsia* from South Australian *B. hydrosauri* ticks collected from *T. rugosa* (sleepy lizard) hosts. These findings identify *B. hydrosauri* as a potential vector of multiple *Rickettsia*. All *T. rugosa* hosts were negative for *Rickettsia* spp. which could be indicative of the limit of detection or could suggest the absence of horizontal transmission from tick to lizard host but does not exclude this form of transmission. This potential absence of horizontal transmission could be explored further through the testing of lizards using serology methods. Further work is also required to characterize the unnamed *Rickettsia* species found.

## References

[1] Dantas-Torres F., Chomel B.B., Otranto D. (2012). Ticks and tick-borne diseases: A one health perspective. Trends Parasitol..

[2] Parola P., Paddock C.D., Socolovschi C., Labruna M.B., Mediannikov O., Kernif T., Abdad M.Y., Stenos J., Bitam I., Fournier P.-E. (2013). Update on tick-borne rickettsioses around the world: A geographic approach. Clin. Microbiol. Rev..

[3] Derne B., Weinstein P., Musso D., Lau C. (2015). Distribution of rickettsioses in oceania: Past patterns and implications for the future. Acta Trop..

[4] Fournier P.E., Raoult D. (2009). Current knowledge on phylogeny and taxonomy of *Rickettsia* spp.. Ann. N. Y. Acad. Sci..

[5] Unsworth N.B., Stenos J., McGregor A.R., Dyer J.R., Graves S.R. (2005). Not only ‘Flinders Island’spotted fever. Pathology.

[6] Abdad M.Y., Cook A., Dyer J., Stenos J., Fenwick S.G. (2014). Seroepidemiological study of outdoor recreationists’ exposure to spotted fever group *Rickettsia* in Western Australia. Am. J. Trop. Med. Hyg..

[7] Murphy H., Renvoisé A., Pandey P., Parola P., Raoult D. (2011). *Rickettsia honei* infection in human, Nepal, 2009. Emerg. Infect. Dis..

[8] Graves S., Stenos J. (2003). Rickettsia honei. Ann. N. Y. Acad. Sci..

[9] Stenos J., Graves S., Popov V.L., Walker D.H. (2003). *Aponomma hydrosauri*, the reptile associated tick reservior of *Rickettsia honei* on Flinders Island, Australia. Am. J. Trop. Med. Hyg..

[10] Dyer J.R., Einsiedel L., Ferguson P.E., Lee A.S., Unsworth N.B., Graves S.R., Gordon D.L. (2005). A new focus of *Rickettsia honei* spotted fever in South Australia. Med. J. Aust..

[11] Parola P., Davoust B., Raoult D. (2005). Tick-and flea-borne rickettsial emerging zoonoses. Vet. Res..

[12] Barker S.C., Walker A.R. (2014). Ticks of Australia. The species that infest domestic animals and humans. Zootaxa.

[13] Vilcins I.-M.E., Fournier P.-E., Old J.M., Deane E. (2009). Evidence for the presence of *Francisella* and spotted fever group *Rickettsia* DNA in the tick *Amblyomma fimbriatum* (acari: Ixodidae), Northern Territory, Australia. J. Med. Entomol..

[14] Lancaster M.L., Gardner M.G., Fitch A.J., Ansari T.H., Smyth A.K. (2012). A direct benefit of native saltbush revegetation for an endemic lizard (*Tiliqua rugosa*) in southern Australia. Aust. J. Zool..

[15] Regnery R.L., Spruill C.L., Plikaytis B.D. (1991). Genotypic identification of rickettsiae and estimation of intraspecies sequence divergence for portions of two rickettsial genes. J. Bacteriol..

[16] Rogall T., Wolters J., Flohr T., Bottger E.C. (1990). Towards a phylogeny and definition of species at the molecular level within the genus *Mycobacterium*. Int. J. Syst. Evol. Microbiol..

[17] Altschul S.F., Gish W., Miller W., Myers E.W., Lipman D.J. (1990). Basic local alignment search tool. J. Mol. Biol..

